# Performance and development trends of ultrasound diagnostic systems in military settings: a review

**DOI:** 10.1186/s13089-025-00458-w

**Published:** 2025-11-04

**Authors:** Chang Lu, He-Jing Huang

**Affiliations:** Department of Ultrasound, Second Affiliated Hospital of Naval Medical University, No. 415 Fengyang Road, Huangpu District, Shanghai, 200003 China

**Keywords:** Battlefield ultrasound, Point-of-care ultrasound (POCUS), Military medical systems, Military medicine, Combat casualty care, Artificial intelligence, Military telemedicine, Tactical medicine

## Abstract

With the evolving challenges of modern warfare, battlefield medical support systems are often required to enhance capabilities in rapid response, flexible deployment, and modular integration. Ultrasound diagnostic systems, appreciated for their portability and ability to provide real-time imaging without ionizing radiation, have been investigated for potential use in early injury screening and rapid assessment in combat and pre-hospital settings. This review provides an overview of representative battlefield-adapted ultrasound systems, such as the Sonosite M-Turbo, Edge II, and GE Vscan Extend, and discusses their reported limitations, including issues with deployment reliability, image quality, operational complexity, and telecommunication capability. Emerging technological directions are explored, including artificial intelligence-assisted diagnosis, multimodal integration, adaptation to extreme environments, and integration with unmanned platforms. Furthermore, a conceptual framework is proposed, focusing on areas such as research and development, standardization, deployment at combat nodes, and training infrastructure, which may contribute to future advancements. The goal is to provide insights that could guide the future development and strategic planning of next-generation tactical medical imaging systems.

## Introduction

As modern warfare increasingly emphasizes information dominance, multi-domain coordination, and asymmetric hybrid operations, the battlefield ​​has been described as involving​​ sudden onset, operational complexity, and ​​a​​ high operational tempo [1–3]. Conventional medical imaging modalities, including X-ray and computed tomography (CT), ​​are constrained by their​​ bulk, deployment difficulties, and radiation risks, which may ​​restrict​​ their applicability for mobile and rapid-response requirements at the front line [4]. In contrast, ultrasound imaging provides ​​a non-ionizing, real-time, and portable alternative​​, and ​​its potential utility in combat casualty care has been reported in multiple studies [5–7]​​, which ​​has coincided with the development of portable ultrasound systems featuring miniaturized and low-power designs​​. ​​The integration of artificial intelligence (AI) has been explored to broaden the application​​ of ultrasound in tactical rescue, far-forward diagnostics, and disaster medicine, ​​with studies suggesting​​ potential adaptability and clinical utility in diverse operational environments [5–7]. Point-of-care ultrasound (POCUS) ​​has been reported to contribute​​ to rapid trauma assessment and battlefield casualty management. ​​For instance​​, even with limited training, military physicians and frontline medical personnel have been able to perform procedures such as the Extended Focused Assessment with Sonography for Trauma (E-FAST) under both combat and simulated conditions, where its use was reported ​​to assist in evacuation prioritization and treatment decision-making​​. ​​Collectively, this body of work provides preliminary evidence for the feasibility of portable ultrasound in military medicine and suggests its potential to support​​ casualty care processes [8]. ​​Nevertheless​​, many current platforms are ​​adaptations of​​ civilian devices and may ​​not be fully optimized for the specific demands of​​ battlefield conditions. Reported limitations include ​​susceptibility​​ to extreme environments, electromagnetic interference, restricted communication bandwidth, and reliance on non-specialist operators. ​​At the same time​​, emerging technologies—such as AI-assisted diagnosis, remote image collaboration, and ​​enhanced​​ operator–system interaction—​​may have the potential​​ to expand the functional scope of battlefield ultrasound systems [7, 9]. Against this background, the present review aims to evaluate the current status and technical limitations of representative battlefield-adapted ultrasound systems, discuss critical operational requirements and usage constraints in tactical contexts, and ​​identify​​ future directions for system-level development and technology integration. The objective is to ​​provide​​ a conceptual framework ​​intended to inform future efforts toward the development of battlefield ultrasound systems with improved efficiency, intelligence, and modularity​​.

## Overview of existing battlefield-adapted ultrasound devices

From a military medical history perspective, the earliest verifiable use of ultrasound within a military health system dates to 1949, when U.S. Navy physician George D. Ludwig at the U.S. Naval Medical Research Institute (NMRI, Bethesda, MD) employed ultrasound to detect gallstones and foreign bodies. This work is ​​frequently cited as an early contribution​​ to the development of medical ultrasonography in a military context [10].​​

The forward deployment of portable ultrasonography​​ during Operation Iraqi Freedom (2003–2004) ​​illustrated its potential clinical utility through several reported cases​​: the U.S. Army Combat Support Hospital (CSH) in Mosul completed more than 400 point-of-care examinations within 6 months; in the British area of operations, handheld Focused Assessment with Sonography for Trauma (FAST) /E-FAST was ​​incorporated into initial assessment in some cases​​ and was reported to influence preoperative decision-making; and a forward support hospital reported point-of-care diagnosis and immediate management of a ruptured ectopic pregnancy. Collectively, these early operational experiences suggested that combat POCUS ​​was feasible and clinically relevant in selected contexts, although systematic evidence for broader scalability was limited​​ [10–13]. In recent years, ​​notable progress has been reported​​ in the development of portable ultrasound systems ​​tailored for​​ military operating environments. Several platforms have been ​​documented as deployed​​ under operational conditions, and their use has been described as ​​contributing to ongoing discussions regarding tactical-level employment frameworks​​ [14, 15]. ​​Against this evolving background​​, the present review seeks to examine ​​representative portable ultrasound systems, focusing on their key performance characteristics, prevailing form factors, and the documented evidence regarding battlefield adaptability​​ [14, 15].

### Common performance features of battlefield-adapted ultrasound devices

Battlefield-adapted ultrasound devices are generally described as sharing several performance features, ​​informed by military environmental standards such as MIL-STD-810G and by reports from field deployments:.

1. Compact and Lightweight Design: ​​Many​​ devices weigh less than 4 kg, which ​​facilitates​​ single-person carriage and ​​allows for relatively rapid​​ deployment ​​in field conditions​​ [16–19].
2. Rapid Boot-Up Capability: Most ​​reported models​​ support cold-start times ​​within approximately 20 s​​, and some ​​have been documented to initiate​​ in as little as 5 s. ​​Such features may assist in enabling​​ timely medical intervention [17].
3. Diagnostic Image Adequacy​​: ​​Although compact in form​​, these devices have been reported to deliver grayscale and Doppler imaging of ​​diagnostically acceptable quality​​, ​​supporting their use in a variety of clinical applications​​ such as battlefield trauma assessment, cardiopulmonary monitoring, and vascular access guidance [18, 20, 21].
4. Rugged Environmental Adaptability: These devices ​​have been reported to​​ meet or partially meet MIL-STD-810G requirements, with resistance to shock, dust, moisture, and temperature extremes. ​​Such characteristics are intended to improve​​ operational reliability under harsh battlefield conditions [7, 14, 18, 21].
5. Multi-Probe and Multi-Mode Compatibility: ​​A number of​​ systems support multiple probes and imaging modes (e.g., B-mode, M-mode, Doppler-mode), ​​allowing for broader evaluation​​ of injuries across different anatomical regions [5, 19, 20].
6. User-Friendly Interfaces and AI-Assisted Functions: Interfaces are ​​frequently designed with relative simplicity​​. In some ​​reported models​​, AI-based diagnostic assistance has been incorporated, ​​with the aim of reducing the cognitive and technical burden​​ on non-specialist operators [6, 20, 22].
7. Telemedicine and Connectivity Capability: ​​Several​​ systems include wireless modules or external communication options, ​​enabling​​ near real-time image transmission and ​​facilitating​​ remote consultation with medical experts ​​when communication infrastructure is available​​ [18–20].

### Representative devices and reported deployment

Although no ultrasound systems are currently designed exclusively for military use, several commercial devices, such as the Sonosite series and the Vscan Extend, ​​were developed for​​ durability and field deployment, ​​as evidenced by​​ their design specifications and testing certifications [18, 23, 24], ​​indicating​​ potential adaptability for military contexts. These systems are typically characterized by a compact and lightweight design, rapid boot-up capability, and long battery life—​​characteristics that support​​ individual carriage and rapid deployment in forward areas. These devices ​​have been documented​​ in field hospitals, telemedicine platforms, and special operations units, where they ​​are primarily used for​​ frontline injury assessment, triage decision-making, and combat casualty care ​​support in selected operational settings​​.

1. Fujifilm Sonosite M-Turbo

The Fujifilm Sonosite M-Turbo is a portable ultrasound system ​​documented in use​​ by the U.S. military’s Forward Surgical Teams (FST) and other tactical medical units. Its design ​​incorporates​​ robust structural shock resistance and ​​references​​ MIL-STD-810G military standards, ​​supporting operation​​ in environments with high humidity, dust, and vibration. ​​Documented clinical applications​​ of this system in military settings include FAST, pneumothorax evaluation, cardiac monitoring, and vascular access guidance, ​​as evidenced in cited operational reports and studies​​ [7, 12, 14, 25–28].

2. Fujifilm Sonosite Edge II

The Fujifilm Sonosite Edge II ​​provides improvements in​​ image quality compared to earlier models such as the M-Turbo, ​​attributable to features including​​ DirectClear™ technology that enhances penetration and tissue contrast. ​​Design elements such as reinforced​​ cable durability and an anti-glare display ​​address operational needs​​ in bright light and other challenging outdoor conditions. Its use ​​has been documented in settings including​​ field hospitals, telemedicine platforms, and special operations units, primarily ​​within defined military medical support roles​​ [18, 21, 29–31].

3. GE Vscan Extend

The GE Vscan Extend includes intelligent assistive features and automated measurement tools ​​designed to facilitate​​ operational workflow ​​by supporting​​ rapid image acquisition and preliminary assessment. Studies ​​have reported its applicability​​ in emergency and critical illness screening, particularly in contexts requiring rapid triage and casualty classification [17, 32, 33]. Based on these characteristics, several reports ​​have suggested its potential suitability for​​ battlefield-related applications, including reconnaissance missions, special operations, and peacekeeping medical modules.

4. Philips Lumify

The Philips Lumify system operates on an Android platform and connects via USB to a smart terminal for control and image storage, ​​a design that enables​​ scalability and wireless data transmission. Its portability ​​has been noted as advantageous​​ in resource-limited environments; ​​published reports describe its use​​ in rear-area hospitals, humanitarian missions, post-disaster relief, and air medical evacuation [19, 32, 34–36]. In tactical contexts, its image transmission capabilities ​​have been described as supportive of​​ telemedicine and diagnostic assistance.

5. Butterfly iQ+.

The Butterfly iQ + is a portable ultrasound system that ​​integrates artificial intelligence (AI) functions​​ and ​​employs​​ a single-probe design capable of operating across a broad frequency spectrum. It incorporates built-in image recognition and automatic labeling algorithms, which are intended to reduce training requirements for frontline personnel and ​​support diagnostic efficiency​​. Although large-scale military deployments ​​have not been reported​​, the system ​​has been noted as a candidate for use​​ in disaster medicine, tactical training exercises, and telemedicine pilot projects, ​​owing to​​ its portability, low power consumption, and reported image quality [6, 37–39]. These attributes ​​have been cited as indicating​​ potential applicability for future integration into battlefield medical support systems.

### Performance comparison and adaptability analysis

Recent portable ultrasound devices ​​demonstrate advancements​​ in portability, imaging quality, and the integration of AI-assisted functions; however, ​​comparative evaluations indicate trade-offs​​ between performance, durability, and potential battlefield applicability. Ruggedized models, such as the Sonosite M-Turbo, ​​have been recognized for​​ greater durability, though ​​reports note limitations related to​​ weight and battery endurance. In contrast, ultra-portable devices like the GE Vscan Extend ​​emphasize​​ mobility, but this ​​can be accompanied by​​ shorter battery life and ​​more constrained imaging performance under certain demanding conditions​​. Systems ​​leveraging​​ commercial smart platforms, such as the Philips Lumify, ​​provide​​ high image quality and advanced telemedicine functionality, yet their limited environmental adaptability ​​has been noted as limiting their applicability​​ in frontline contexts. Emerging solutions that incorporate AI and multifunctionality, such as the Butterfly iQ+, ​​have reported challenges​​ related to power management and operational stability, ​​which warrant further evaluation before consideration for sustained field use​​. A structured comparison of these strengths and limitations ​​is provided in​​ Table 1, ​​outlining their reported suitability​​ across different military operational settings.

**Table 1:** Performance characteristics and deployment features of representative ultrasound systems in military applications

Device name	Launch date	Basic physical parameters			Imaging modes and functions		Environmental adaptability and standards		Structural and design features	Usability and deployment efficiency		Connectivity and remote support capabilities		Clinical applications		Qualitative assessment dimensions for operational suitability			
		Weight	Startup time	Battery life	Imaging modes	Key features	Feature description	Standard reference								Strengths	Limitations	Battlefield suitability	
Sonosite M-Turbo [5, 12, 15, 26–28, 40]	2007	~ 3.4 kg	< 20 s	~ 2 h (varies with imaging mode and display brightness)	B, M, Doppler modes	1. Support for E-FAST 2. Needle-enhanced display	1. Drop-tested from approximately 0.9 m 2. Documented use in operational environments 3. High resistance to electromagnetic interference	1. Designed with reference to military-grade standards 2. Full compliance with MIL-STD-810G not fully documented	1. Enclosed housing protects both screen and keyboard 2. Backlit keyboard 3. Compact, ruggedized structure contributing to durability	1. Short cold start time facilitates rapid deployment 2. Documented use in tactical frontline contexts		Limited wireless functionality (Digital Imaging and Communications in Medicine [DICOM] and Wi-Fi support not publicly disclosed)		Reported image quality considered suitable for battlefield trauma evaluation, cardiopulmonary monitoring, and vascular access guidance		Rugged design, reported reliability of imaging performance	Relatively heavy, short battery life	Reported as suitable for frontline trauma care, though limited by operating endurance	
Sonosite Edge II [29–32]	2016	~ 4.1 kg	< 25 s	~ 2 h (varies with imaging mode and display brightness)	B, M, Doppler modes	1. Support for E-FAST examination 2. Image enhancement 3. Enhanced needle visualization 4. Real-time guidance 5. AI-assisted image acquisition and interpretation (features reported as under development)	1. Documented use in operational environments 2. Reinforced structure; liquid-resistant and anti-glare screen	1. Full compliance with MIL-STD-810G not fully documented 2. Reported compliance with the International Electrotechnical Commission (IEC) 60601-1-2:2014 standard for Electromagnetic Compatibility (EMC)	1. Reinforced chassis design 2. Anti-glare screen 3. Liquid ingress protection 4. Interface noted as user-friendly in operational settings	1. Rapid startup capability 2. Interface described as facilitating operation in complex environments		1. Supports DICOM communication 2. Wi-Fi enabled		1. Reported application in rapid diagnosis within complex environments 2. Supports trauma evaluation and vascular access guidance 3. Image quality and functions documented as applicable in higher-complexity scenarios		Improved image quality, reported durability enhancements	Relatively bulky, short battery life (~2 h)	Reported as suitable for field hospitals and special operations units	
GE Vscan Extend [32, 33]	2017	~ 500 g	~ 40 s	~ 1 h	B, Doppler modes	1. Dual probe configuration: linear + phased array 2. Guidance for rapid FAST examination 3. Automatic bladder volume calculation 4. AI-assisted lung diagnostics	1. Operating temperature 0–40℃ 2. Humidity 15–90% (non-condensing) 3. Pressure 620–1060 hPa	1. Probe protection rating: IPX7 2. Main unit protection level IP33 3. Full compliance with MIL-STD-810G not fully documented	1. Pocket-sized ultra-portable design 2. Smartphone-like user interface 3. Reported as designed for rapid screening and portability	1. Rapid deployment capability 2. Simplified interface 3. Reported as suitable for frontline screening		1. Supports wireless image sharing 2. Reported as designed for mobile healthcare use		1. Documented use in frontline and mobile medical applications 2. Reported utility in resource-limited environments 3. Enables rapid basic evaluation of pulmonary and urinary systems		Ultra-portable design, dual probe, ease of use	Short battery life (~1 h), limited imaging modes	Reported as appropriate for rapid triage in resource-limited environments	
Philips Lumify [32, 36, 41]	2015	Probe weight < 136 g (ultra-lightweight)	Dependent on external mobile devices	Dependent on external mobile devices	B, Doppler modes	1. Single probe with broad frequency range (1–10 MHz) 2. AI-assisted features: image acquisition, B-line detection, EF estimation 3. Partial 3D scanning support 4. Remote collaboration and telemedicine features	Impact and vibration tested from approximately 1.2 m	1. Probe protection rating: IP47 2. Reported system-level adaptability dependent on paired smart device 3. Compliant with CISPR 11 Class B and IEC 60601-1-2 EMC standards 4. Incorporates certain military-grade hardware components (reported) 5. Full compliance with MIL-STD-810G not fully documented	1. Probe connects via USB directly to Android/iOS devices 2. Lightweight, modular design noted as enhancing portability 3. Supports wireless charging (device-dependent)	1. Console-free operation utilizing commercial smart device interface 2. Plug-and-play functionality reported as supporting simple operation and rapid activation		1. Supports wireless image transmission 2. Reported as enabling remote collaboration and teleconsultation 3. Compatible with mobile telemedicine systems		1. Documented use in rear-area medical care and remote diagnostics 2. AI-assisted features reported as potentially enhancing tele-ultrasound applications 3. More frequently reported in stable environments than in high-intensity frontline operations		High image quality, AI features, telemedicine-ready	Performance dependent on host smart device, limited inherent ruggedization	Reported as well-suited for rear-area hospitals and telemedicine platforms	
Butterfly iQ+ [12, 18, 26, 39, 42]	2020	Probe weight ~ 300 g (lightweight)	Dependent on external mobile devices	≥ 2 h (B mode); requires external power for recharging	B, M, Doppler modes	1. Single probe with full frequency range (1–10 MHz) 2. Needle visualization tool (needle tracking) 3. Automatic cardiac ejection fraction (EF) estimation 4. Automatic bladder volume estimation	1. Documented use in challenging environments 2. Rated resistance to dust and water (IP67)	1. Protection rating: IP67 2. Full compliance with MIL-STD-810G not fully documented	1. Compatible with iOS and Android platforms 2. Connects to mobile devices via USB 3. Highly integrated, modular all-in-one design	1. Rapid startup and integration reported as supporting deployment in field scenarios 2. Intuitive interface noted as reducing barriers for non-specialist use 3. Offline functionality reported as enabling use in remote or resource-limited settings		1. Supports wireless image transmission 2. Reported as enabling remote scanning assistance and diagnostics 4. Compatible with hybrid wireless/wired communication systems		1. Documented field use (e.g., Ukraine conflict) 2. Reported application in rapid trauma triage, vascular access guidance, and cardiopulmonary/urinary assessment 3. AI integration reported as potentially improving usability for non-professionals 4. Proposed applications include primary care and tactical telemedicine		Full-spectrum probe, AI support, lightweight design	Limited battery endurance, connectivity dependent on external device	Reported as an emerging solution for tactical drills, telemedicine pilots, and documented field applications	

### Clinical evidence on the application of military ultrasound

Current evidence suggests that the clinical use of ​​ultrasound in military settings​​ has been examined through both prospective and retrospective studies. Savell et al. [8] ​​conducted a literature review documenting the use of point-of-care ultrasound (POCUS) for​​ diagnostic support, procedural guidance, and patient monitoring by multidisciplinary personnel in combat and austere environments; reported applications included ​​the E-FAST exam​​, vascular access, and decision-making during cardiac arrest. In a retrospective analysis of 401 examinations performed at a forward-deployed hospital during Operation Iraqi Freedom, Rozanski et al. [11] reported that ​​the use of portable ultrasound​​ was associated with expanded diagnostic capacity, fewer unnecessary evacuations, and improved efficiency of frontline medical care.

Prospective studies have further explored these observations. In a randomized crossover trial, Salazar et al. [18] reported that after brief training, combat medics achieved ​​notable diagnostic accuracy​​ in E-FAST examinations using both novel and conventional portable ultrasound devices; their findings suggested that device miniaturization and interface optimization ​​did not adversely affect​​ diagnostic performance. Similarly, Cazes et al. [43] observed that after an accelerated training program, military physicians were able to perform sonographic assessments for FAST, bladder, aorta, and pleural evaluations following a limited number of practice sessions. Perrier et al. [44] additionally reported that after short-term training, junior military physicians demonstrated improved diagnostic accuracy in 73% of cases, ​​highlighting the potential of POCUS as a decision-support tool for​​ less experienced providers.

The ​​use​​ of ultrasound has also been documented in mass-casualty and resource-limited settings. In a multicenter retrospective study across sites in Africa and the Middle East, Dubecq et al. [45] reported that among 325 casualties, ultrasound confirmed diagnoses in 74 (23%), ruled out an initial suspicion in 205 (63%), and changed surgical prioritization in 140 (43%); these observations were associated with subsequent triage and treatment decisions. Similarly, during the Second Lebanon War, Beck-Razi et al. [46] reported that FAST had an overall diagnostic accuracy of 93.1% in wartime emergency triage and was used to inform decisions on surgery, imaging, and observation. ​​However, the generalizability of these findings may be constrained by the retrospective designs and the high-acuity wartime and disaster settings.

Building on these findings, mission reports from recent multinational humanitarian and non-combat operations provide further case examples of portable ultrasound system utilization. During Mission Harmony-2018, the Chinese Navy hospital ship Peace Ark carried two portable ultrasound devices—the Wisonic Clover60 and the Mindray M7—in addition to a console system. Of the 5277 examinations performed, 3124 (59.2%) were conducted using portable devices across triage, bedside diagnostics, and outreach medical services, as reported in the mission records [47]. In the 2019 joint U.S.–Brazilian humanitarian assistance mission aboard the Carlos Chagas hospital ship, the SonoSite iViz handheld ultrasound (P21v, L38v) was used in 24 outpatient cases among 814 patients; according to mission notes, clinical management was changed or confirmed in 10 cases (42%), and a lower number of referrals was recorded. ​​However, due to the small sample size (*n* = 24), these findings should be interpreted with caution​​ [48]. Similarly, during the 2010 Haiti earthquake response, the New Mexico Disaster Medical Assistance Team ​​piloted​​ the Signos handheld ultrasound (Signostics, USA) for their team’s first deployment use; examinations were reported to affect clinical management decisions in 36 cases (70%), aiding in triage, obstetric evaluation, vascular assessment, and procedural guidance [49]. ​​It is important to note that this was a pilot deployment in a high-acuity disaster setting, which may limit the generalizability of the reported findings.

Across Europe, reports describe the incorporation of portable ultrasound into disaster response and prehospital emergency systems. A 2024 evaluation of a UK helicopter emergency medical service (HEMS) ​​program reported the routine use of​​ handheld point-of-care ultrasound for cardiac and trauma assessment, ​​with comparable image quality noted between​​ physicians and paramedics ​​and supported by​​ cloud-based archiving and governance frameworks [50]. Similarly, within the Franco-German prehospital emergency model, portable ultrasound ​​is described as aiding​​ early diagnostic assessments and informing transfer decisions under the physician-led “on-site diagnosis–stabilization–transport” paradigm [51].

From a technical standpoint, contemporary handheld units—some models weighing approximately 260 g—offer features such as real-time color Doppler imaging, Wi-Fi connectivity, and multi-probe compatibility. These features are intended to support operation in austere environments, although practical use may be constrained by network availability, battery runtime, and environmental conditions. Compared with high-end cart-based systems, handheld devices generally provide lower image resolution and shorter battery life [6]. Nonetheless, field studies have reported that, for selected trauma and musculoskeletal applications, portable ultrasound had diagnostic performance comparable with console-based systems in small cohorts; a few studies also reported concordance with MRI findings in specific contexts [52].

In summary, evidence from prospective and retrospective studies, alongside reports from humanitarian operations and non-combat missions, suggests the feasibility and potential utility of portable ultrasound in military and disaster medicine. Documented use spans forward-deployed hospitals, mass-casualty incidents, naval missions, aeromedical evacuation, and disaster relief operations. Devices such as the Mindray M7, Wisonic Clover60, SonoSite iViz, Signos handheld ultrasound, and SonoSite 180 have been employed to aid triage, bedside assessment, and procedural guidance, with some studies reporting associated changes in clinical management or referral patterns [47–49, 52, 53]. ​​Given the heterogeneity of settings, devices, and study designs, generalizability remains limited.​

## Operational challenges in battlefield use

While portable ultrasound has ​​potential applications​​ in battlefield medicine, its practical use is ​​constrained by device-level limitations and the inherent complexities of combat environments​​. ​​Constraints stem from heterogeneous injury patterns, austere conditions, and resource-limited workflows. A system-level assessment​​ of these ​​operational constraints is needed to define key performance requirements and guide​​ the development of next-generation, ​​battlefield-ready​​ ultrasound systems.

### Harsh battlefield environments and high durability requirements

Battlefield environments often involve extreme conditions, including high temperatures, humidity, sandstorms, ​​precipitation (including snow)​​, severe vibrations, and electromagnetic interference. These conditions impose ​​stringent requirements for mechanical robustness, ingress protection, electromagnetic compatibility, and power resilience​​ in ultrasound systems [54, 55]. Heiner et al. [55] noted that although modern handheld devices offer advantages in portability and low weight, reliability under severe vibrations, ​​adverse weather​​, and unstable power supplies remains a concern. Similarly, Feletti et al. [54] reported that most commercially available models are not explicitly designed for extreme environments, with ​​frequently cited​​ issues such as battery performance degradation, reduced ​​display readability​​, and wear at probe interfaces. While newer platforms (e.g., Butterfly iQ+ and Philips Lumify) incorporate features such as image transmission and AI-assisted functions, ​​available reports indicate that protective characteristics—including impact resistance and overall ruggedization—​​ may still be inadequate for high-intensity combat or ​​prolonged​​ field deployment, particularly ​​in the absence of​​ fully shock-resistant structural designs. Therefore, ​​environmental hardening​​ and structural reliability remain ​​critical priorities for innovation​​ in the development of tactical ultrasound systems.

### Strong demand for rapid deployment and one-button operation

Trauma care in battlefield settings is often time-critical, with a narrow intervention window—often described as the “golden 10 min”—during which rapid ultrasound imaging and preliminary ​​assessment​​ are often required [18, 56]. Howard et al. [56] noted that faster intervention has been associated with lower mortality among combat casualties, underscoring the need for ultrasound systems that enable rapid deployment and immediate use. ​​In line with this,​​ Nelson et al. [57] reported that devices intended for battlefield and disaster environments should support ​​immediate usability at power-on​​ and offer simplified user interfaces to facilitate ​​time-sensitive decisions​​. ​​Operator experience and training levels also influence examination timeliness.​​ Shi et al. [58] observed that many mobile and tele-ultrasound initiatives operate under training constraints and that conventional interfaces can present a steep learning curve, with implications for clinical efficiency. Breunig et al. [59] documented ​​variability in learning curves​​ across anatomical targets—with cardiac imaging among the most challenging and tubular-organ imaging more accessible—highlighting the importance of interface simplification and intelligent user guidance, particularly for ​​less-experienced operators​​. Addressing these needs, Milletari et al. [60] proposed integrating ​​AI-driven​​ image presets, real-time ​​acquisition feedback​​, and one-button workflow initiation as ​​potential approaches to enhance portable ultrasound systems in low-experience contexts​​. In summary, design priorities for next-generation battlefield ultrasound include ​​improved cold-start performance, minimal interaction burden (e.g., one-button “boot-to-scan”), and embedded guidance and automation​​ to support rapid, effective use under operational stress.

### Complex injury types and the need for balanced image quality and clinical adaptability

Battlefield injuries are ​​diverse​​ and may include closed abdominal injuries, pneumothorax, pericardial tamponade, and fractures with vascular damage, which may, ​​in some circumstances, require multimodal imaging for a more comprehensive assessment​​ [52]. Gao et al. [6] ​​reported the use of portable ultrasound to evaluate​​ abdominal hemorrhage, fractures, hemothorax, and pericardial effusion, and ​​observational evidence suggests potential value for supporting​​ triage during mass-casualty events. ​​At the same time, given engineering constraints​​ such as power consumption and device size, some portable systems ​​involve a trade-off between​​ penetration and resolution, which ​​in specific applications may reduce visualization of​​ deep structures or small-volume hemorrhage. Baugher et al. [61] found that handheld devices ​​scored lower on image-quality metrics than​​ cart-based systems, with more pronounced differences in deeper regions (e.g., liver and kidney). ​​Under the evaluation conditions reported by​​ Merkel et al. [62], some devices provided acceptable imaging of targets such as the inferior vena cava (IVC) and intra-abdominal fluid, whereas performance was relatively limited for higher-resolution tasks such as assessing liver lesions or gallbladder wall stratification. Lucas et al. [63] highlighted that probe frequency directly affects the balance between resolution and penetration; higher frequencies enhance superficial detail but diminish depth performance, ​​necessitating trade-offs when multiple anatomical layers must be assessed​​. Additionally, some ​​early-generation or lower-cost​​ portable devices may lack or offer limited advanced capabilities (e.g., harmonic imaging, color Doppler), which can constrain adaptability when hemodynamic or contrast-like information is needed. Therefore, ​​based on the current technological landscape​​, the development of next-generation battlefield ultrasound systems ​​could prioritize achieving a better balance between image quality and clinical adaptability. This includes advancing miniaturization while explicitly managing inherent performance trade-offs, and incrementally integrating multimodal capabilities, which may be necessary to enhance device applicability across the complex and varied scenarios encountered in operational environments​​.

### Communication limitations and remote collaboration barriers

Wireless handheld ultrasound devices ​​offer​​ portability and rapid image acquisition for battlefield medicine, yet their use in combat environments presents distinct security and communications challenges [38]. Conventional wireless protocols (e.g., Wi-Fi, Bluetooth) may be vulnerable to interception and electromagnetic interference; ​​in the absence of robust encryption and hardened implementations​​, reliability may be reduced ​​under high-threat or contested electromagnetic conditions​​ [64]. While 5G can provide high bandwidth and low latency for real-time transmission and teleconsultation, its applicability depends on infrastructure availability, coverage, and cybersecurity risk management [65]. Wi-Fi is typically more accessible but can be constrained by short range and susceptibility to interference, and forward-deployed settings are often characterized by low bandwidth or blackout conditions that affect image transfer and remote diagnosis [64].

Field reports further illustrate these constraints. Rosser et al. [66] showed that telemedicine was feasible at 12–28 kbps using compression and transmission strategies, though image resolution, latency, and disconnections were limiting factors. Cermack et al. [67] noted that, although satellite communications provide broad coverage, power, cost, and encryption requirements may restrict high-quality imaging and physiologic data transfer in extreme conditions. Kim et al. [38] observed that—despite LTE- and Wi-Fi-enabled transmission—devices such as the Butterfly iQ+, ​​at the time of their evaluation​​, did not include standardized compression, end-to-end encryption, or offline AI functions, potentially reducing efficiency in battlefield and disaster scenarios. Although AI may assist image acquisition and preliminary interpretation in resource-limited settings, secure transmission, interoperability, and validation remain ongoing concerns.

In light of these documented limitations, advancing resilient communication for battlefield ultrasound remains a key challenge. Next-generation systems would therefore need to incorporate secure, encrypted communications; low-bitrate, error-resilient compression; and adaptive transmission strategies to function more reliably in low-bandwidth environments. The integration of offline or edge-deployable AI analysis would mitigate dependence on unstable network connections. Ultimately, seamless interoperability with satellite and tactical communications networks will be necessary to support reliable, secure, and continuous collaboration under the austere and high-threat conditions characteristic of the battlefield.​.

### Tactical portability and energy management issues

Military ultrasound devices must balance ​​portability with energy endurance​​. Stawicki et al. [68] reported ​​deployment of​​ portable systems in extreme environments through miniaturization, battery-powered operation, and ​​ruggedization​​, with some units weighing under approximately 5 kg and ​​demonstrating​​ shock resistance and environmental adaptability ​​in their evaluations​​. Nevertheless, tactical applications continue to encounter mobility constraints. Nelson et al. [57] found that device weight, ​​probe cabling​​, and ​​power-supply design​​ can ​​materially affect​​ frontline mobility. Kim et al. [38] and device manuals [27, 29, 33, 36, 39] indicated that, ​​in some models​​, battery life (< 3 h), slow charging, ​​non–hot-swappable batteries​​, and limited backup options may be insufficient for continuous, high-demand operations. Stawicki et al. [68] further emphasized that, in settings without stable power, quick-change batteries, modular architectures, and low-power optimization may be important for ​​sustaining capability​​.

In light of these documented constraints, the development of next-generation systems will need to focus on addressing limitations in weight, energy management, and hardware design. This would entail incorporating lightweight form factors, robust and snag-resistant probe cabling, and efficient energy solutions—such as hot-swappable batteries and AI-assisted low-power optimization—to extend operational endurance. A modular architectural approach would enhance flexibility and sustainability in the field. Collectively, these features would therefore be necessary to support sustained diagnostic tasks under the high-mobility and power-limited conditions characteristic of battlefield medicine.​.

## Future trends and technological directions

As battlefield medical systems evolve toward greater ​​autonomy, connectivity,​​ and forward deployment, ultrasound platforms are being explored not only as imaging tools but also as potential intelligent nodes within operational medical networks. In light of current application limitations and ongoing technological ​​developments​​, future ​​research and development could prioritize​​ the following directions, while explicitly addressing persistent constraints related to data quality, interoperability, security, and clinical validation.

### Intelligence and AI-assisted diagnosis

AI is ​​widely discussed as a driver of​​ the intelligent evolution of battlefield ultrasound devices. According to ​​the​​ NATO Science & Technology Trends 2023–2043 report, AI ​​is identified as​​ one of the top disruptive strategic technologies, with military medical applications ​​reported to focus on​​ image processing, decision support, and cross-platform collaboration [1]. In this context, the integration of AI with ​​big-data​​ analytics and robotic systems ​​may influence​​ battlefield diagnostics ​​by augmenting​​ acquisition, triage, and workflow support. Over time, ultrasound devices ​​could evolve from primarily imaging endpoints toward​​ AI-enabled, data-integrated components of closed-loop decision support, ​​subject to meeting critical requirements for secure transmission, model generalizability, human-in-the-loop oversight, and prospective validation. This trajectory could strengthen​​ coordination between frontline medical elements and tactical systems, ​​contingent on maintaining rigorous safety and performance controls​​.

AI in battlefield ultrasound is being explored as contributing to a “technology–function–value” ​​conceptual framework​​. At the technological level, Liu et al. [69] demonstrated that deep learning can reach accuracy levels comparable to clinical standards in image classification (area under the receiver operating characteristic curve [AUC] = 0.92), detection (accuracy 91.5%), and segmentation (Dice 94.5%). The AI-LUS algorithm developed by Fiedler et al. [70] ​​achieved​​ 92.1% sensitivity and 80.2% specificity in pneumothorax screening, with a single scan time of less than 15 s. At the functional level, three key tasks have been identified: (1) automatic identification of the FAST region to assist in effusion screening [69]; (2) intelligent anomaly alerts (e.g., “possible pneumothorax”) to enhance diagnostic performance [70]; and (3) local AI processing to support offline inference, ​​potentially improving​​ diagnostic reliability in low-bandwidth environments [69, 70]. In terms of practical value, portable ultrasound combined with AI algorithms, supported by robotic platforms, ​​may facilitate​​ more standardized image acquisition and support rapid preliminary assessment, ​​with the potential to improve​​ triage efficiency [6]. Levy et al. [71] applied the DenseNet121 model to 6,608 battlefield FAST images, achieving an accuracy of 98.0%, sensitivity of 94.0%, and specificity of 100% for hemorrhage detection, and reported high image quality; such performance ​​could assist​​ less experienced operators in improving diagnostic consistency. Kim et al. [38] highlighted that AI-supported POCUS ​​has the potential to reduce​​ operational barriers and ​​enhance​​ efficiency and consistency in low-resource settings, particularly in small teams, remote forward positions, and isolated mission scenarios.

Despite these advances, current applications of AI in ultrasound still face important limitations. First, most models are trained on relatively small, single-center datasets with limited diversity, which ​​could limit​​ their generalizability across different populations, devices, and clinical contexts [38, 69]. Second, the lack of standardized imaging protocols, data formats, and interoperability among portable ultrasound systems ​​may pose​​ challenges for cross-platform deployment in battlefield conditions [6, 38, 71]. Third, existing AI functions ​​remain​​ relatively limited, focusing primarily on image optimization, annotation, or basic lesion detection, while autonomous diagnostic reasoning and complex task execution are not yet well developed [69–71]. Fourth, computational and communication constraints pose additional barriers: many algorithms ​​require​​ stable power and bandwidth, whereas battlefield environments are typically characterized by low-resource settings [6, 38]. Finally, issues of data privacy, ethical governance, and insufficient operator training may further constrain clinical applicability and user trust [38, 70]. Addressing these challenges ​​will be critical for​​ advancing AI-enabled battlefield ultrasound from descriptive assistance toward more robust, autonomous, and field-ready diagnostic support.

In conclusion, although current AI applications in ultrasound still face limitations in data diversity, standardization, and field validation, AI ​​is facilitating​​ a gradual transition of battlefield ultrasound from a conventional imaging modality toward an intelligent diagnostic platform. Future systems ​​should therefore focus on advancing​​ key capabilities such as intelligent guidance, automatic interpretation, and offline AI processing. ​​Such advances would enhance​​ battlefield medical intelligence and ​​strengthen​​ the diagnostic capabilities of operational military medical systems.

### Multifunctional capabilities and system-level integration

Battlefield medical devices are increasingly being developed with trends toward lightweight designs and multifunctional integration to address the demands of frontline treatment in highly mobile and complex environments. Future ultrasound systems could likely enhance imaging performance while also incorporating physiological monitoring, rapid testing, and communication modules, potentially forming an integrated “single-soldier medical terminal” platform [72].

Dias et al. [72] noted that wearable health devices (WHDs) ​​could have​​ considerable potential for use in battlefield and emergency environments, particularly for continuous monitoring of vital signs during high-intensity combat. Advanced WHDs currently integrate sensors for electrocardiogram (ECG), oxygen saturation (SpO₂), respiratory rate, and skin temperature, and employ wireless communication for real-time data transmission and remote analysis. Spicher et al. [73] demonstrated, through 5G edge computing platforms, that the integration of 5G and edge computing with wearable sensors (e.g., smart textiles, ECG) can provide low latency (< 110 ms) and high transmission reliability, which ​​could provide​​ a technological foundation for future tactical medical system integration. Vega et al. [32] further emphasized that integrating ultrasound with wearable sensors, combined with cloud computing and AI algorithms for remote operation and diagnosis, ​​could support​​ the development of an intelligent closed-loop system linking physiological monitoring, image analysis, and intervention. Anderson et al. [7] pointed out that portable ultrasound devices deployed in battlefield environments may need to integrate imaging, monitoring, and communication functions while meeting tactical requirements for lightweight design and durability (shockproof, waterproof, dustproof). A comparison of representative systems shows: (1) Butterfly iQ + uses an integrated probe-terminal connection for convenient deployment; (2) Philips Lumify provides high image quality and strong compatibility; and (3) GE Vscan Extend is reported to have long battery life and robust durability, which may make it suitable for tactical scenarios such as E-FAST [17, 74].

In conclusion, the development of future battlefield ultrasound systems ​​is likely to involve the integration of​​ physiological monitoring (e.g., ECG, SpO₂, blood pressure), rapid testing (e.g., blood glucose, electrolytes), wireless communication (e.g., 5G, satellite links), and local AI analysis modules. Such integration ​​may facilitate​​ low-latency remote collaboration and system-level diagnostic feedback. Development efforts ​​could emphasize​​ intelligent multi-module integration and task-level collaboration, with the aim of improving battlefield medical response efficiency and ​​supporting​​ more continuous, high-quality operational medical care.

### Strengthening adaptability to extreme tactical environments

Future combat environments are becoming increasingly diverse, with extreme conditions such as high altitude, freezing temperatures, deep-sea settings, and uninhabited areas likely to place greater demands on the adaptability of battlefield medical equipment. As a commonly used tool for frontline medical care, ultrasound systems ​​will need to enhance​​ their robustness and operational stability under complex environmental conditions [72, 75].

Speicher et al. [76] noted that wearable ultrasound systems are improving their reliability in high-humidity, dusty, and dynamic environments through high-sealing designs, flexible printed circuit board (PCB) structures, and low-power wireless data links, which may support progress toward “wear-and-collect” functionality. Dias et al. [72] emphasized that devices should aim to provide robust dustproof and waterproof protection and be capable of withstanding extreme temperatures, humidity, and mechanical shock, to help support stable operation during long-term, high-intensity missions. In terms of probe design, Speicher et al. [76] compared six handheld ultrasound devices and reported that integrated probes (e.g., Butterfly iQ+) are easy to operate and were generally considered favorably by experts, although 89% of experts indicated that image quality required further improvement. Wireless probes (e.g., Vscan Air) are highly portable, but some users reported connectivity instability. Ultra-light probes (e.g., Mindray, 198 g) may improve operational comfort, whereas heavier devices (e.g., Kosmos, 280 g) can sometimes reduce operational flexibility. Regarding power supply, the focus ​​should be placed on​​ autonomy and environmental adaptability of the energy system. Shadvar et al. [77] highlighted that off-grid solar systems in combat zones or remote areas ​​have shown potential for reliability and cost-effectiveness​​, providing potential support for critical equipment. Sohail et al. [78] reviewed energy harvesting technologies (e.g., piezoelectric, thermoelectric, photovoltaic) that have been explored in low-power medical devices, which can harness energy from body motion, temperature gradients, or natural light, and may be suitable for high-mobility and energy-limited scenarios. Tactical medical units integrated with solar panels and wearable energy storage systems, combined with military-standard shockproof interfaces, have been reported to support extended operation times (e.g., up to 48 h) in frontline medical support. Ultrasound devices, as rapid diagnostic tools, also rely on efficient power supply systems to help maintain reliable operation. In tactical conditions, lightweight energy storage modules, solar energy support systems, and energy harvesting solutions ​​could be considered to support​​ stable functionality and deployment capability in off-grid environments.

In conclusion, ​​developing next-generation battlefield ultrasound systems with improved environmental adaptability will be essential. This includes incorporating​​ lightweight yet robust designs, enhanced functional integration, low-power wireless communication capabilities, and efficient sustainable energy solutions. ​​Progress in these areas would enhance​​ diagnostic efficiency and ​​support more effective​​ frontline medical care across diverse operational conditions.

### Enhanced communication and remote collaboration capabilities

In the context of multi-domain integration and high-tempo warfare, ​​information-driven capabilities are increasingly critical for battlefield resilience​​. Specifically, the efficient transmission and intelligent processing of diagnostic images and decision-making data in battlefield medical systems ​​have been identified as crucial​​ for improving frontline responsiveness and enhancing coordination with rear support [79–81]. Pamplin et al. [81] emphasize that medical information chains, as crucial nodes within a broader battlefield network, ​​could benefit from​​ intelligent, distributed architectures to establish more continuous and reliable data pathways for operational medical decision-making. Peterson et al. [79] empirically demonstrated that MPEG-4 compression can achieve high compression ratios for CT data while maintaining diagnostic quality, thereby supporting the feasibility of image transmission in low-bandwidth environments. Additionally, early field studies on low-bandwidth telemedicine ​​have indicated that​​ remote medical consultations can be conducted over links as low as 10 kbps using appropriate compression and transmission strategies, although resolution and latency ​​are often compromised​​ [66].

Regarding collaborative tools, Butterfly Network’s AR-based remote guidance ​​is being explored for its potential to enhance​​ ultrasound operation efficiency between frontline personnel and remote experts [82]. AI-supported POCUS systems ​​have been shown to improve​​ image interpretation consistency and operational efficiency in resource-limited environments, which ​​may support​​ remote collaboration in battlefield scenarios [38]. Furthermore, Votel et al. [80] noted that in gray-zone conflicts and hybrid warfare, cross-domain information integration and the construction of communication links ​​are important​​ for maintaining operational resilience. RAND’s research indicates that tactical satellite communications ​​were used to support​​ high-tempo image and information transmission during the Ukraine conflict, although commercial LEO links faced global outages, ​​highlighting the need for​​ multi-link redundancy and rapid switching [83, 84]. As for communication links, public Wi-Fi and Bluetooth are generally considered ​​vulnerable as primary connections​​ on the frontline due to security and interference concerns. Instead, encrypted tactical communications, such as MANET, tactical 5G, or SATCOM, ​​have been proposed for prioritization​​, with end-to-end encryption and strong authentication protocols ​​identified as critical requirements​​ [64, 85, 86].

Based on these tactical communications, ​​the implementation of​​ multi-link redundancy, automatic switching, and adaptive bitrate ​​could mitigate​​ disruption risks associated with the instability of commercial LEO satellites and partially address interference issues [83, 84]. Additionally, the U.S. Department of Defense’s 5G telemedicine and AR trials ​​demonstrated that​​ low-latency remote guidance was achievable under controlled tactical/private 5G conditions, ​​indicating the feasibility of​​ remote collaboration in battlefield ultrasound [87]. To enhance interoperability and bandwidth efficiency, edge devices ​​could be configured to transmit​​ keyframe/fragment-based data through DICOMweb and HL7 FHIR structured measurement summaries, which ​​could improve efficiency by prioritizing the transmission of​​ key points before sending full datasets [89, 90].

In conclusion, future battlefield ultrasound systems ​​are likely to evolve toward​​ greater integration and intelligence, potentially incorporating a composite network architecture of “near-range MANET + SATCOM as a fallback + enhanced private/tactical 5G,” along with low-bitrate/layered compression, adaptive transmission (with storage-forwarding when necessary), end-to-end encryption, zero-trust security frameworks, and AR collaboration and offline/edge AI analysis capabilities. ​​This integrated approach could support​​ the development of a closed-loop system of “frontline acquisition → intelligent analysis → remote review → command decision-making,” and ​​could help address​​ the requirements for rapid diagnosis and collaborative decision-making in multi-domain, high-mobility operations [64, 66, 79–83, 87, 88].

### Adaptation to autonomous and unmanned systems

There is a growing demand for rapid, low-risk, and highly mobile medical support on future battlefields, with casualty location and initial screening ​​increasingly oriented toward​​ unmanned platforms and autonomous systems. As a core imaging tool, portable ultrasound systems ​​will need to be adapted for integration​​ with unmanned technologies to support intelligent injury detection and tactical decision-making [24, 91].

Monfaredi et al. [91] reported that robot-assisted ultrasound allowed remote physicians to control ultrasound operations via image guidance and force feedback, which ​​was shown to improve imaging quality in their study​​. Ye et al. [92] explored the feasibility of 5G-enabled robotic remote ultrasound systems in complex clinical environments, ​​demonstrating​​ real-time control, AI-based image analysis, and reduced exposure risks, which suggests potential applicability in battlefield contexts. Remondelli et al. [93] introduced the concept of “digital twin-driven battlefield injury management,” which aims to integrate real-time sensor data, AI models, and unmanned systems to develop a prototype of an ‘intelligent injury map’ for ​​supporting​​ tactical command and casualty evacuation decisions. This approach would ​​require​​ ultrasound systems to incorporate standardized communication protocols, efficient data interfaces, and AI compatibility.

Several technological pathways are currently under investigation: (1) unmanned ground vehicles (UGVs), quadruped robots, and low-altitude unmanned aerial platforms equipped with ultrasound modules ​​are being explored for performing​​ automated ultrasound scanning tasks in high-risk areas [91, 92]; (2) with the support of 5G communication, remote physicians ​​can control​​ ultrasound probes in real time to perform imaging assessments, ​​thereby reducing​​ frontline exposure risks [92]; and (3) integration of continuous data transmission (CDT) with AI-based analytic techniques ​​has been proposed to develop​​ real-time battlefield injury maps, which ​​may support​​ tactical tasking and medical resource allocation [93].

In conclusion, future portable ultrasound systems ​​are likely to evolve toward​​ intelligent and autonomous platforms, with emphasis on advances in lightweight modular design, high-degree-of-freedom robotic arm integration, and standardized interface development. By incorporating 5G communication, AI-based analysis, and digital twin-enabled architectures, these systems ​​could perform​​ remote imaging and injury mapping on unmanned platforms, which ​​would provide​​ more efficient and accurate decision support in battlefield medicine.

## Discussion & recommendations

Although ultrasound diagnostic systems have made notable progress in military applications and have been reported to provide value in some combat-related missions, existing devices still exhibit important limitations in equipment maturity, operational adaptability, intelligent functionality, and system integration. These limitations ​​may limit their ability to fully address​​ the medical support needs of modern battlefields.

### Key limitations and shortcomings

Based on the analysis of the parameters and applications of the five representative devices discussed above, ​​these devices demonstrate​​ favorable portability, diagnostic imaging performance, and multimodal functionality. However, ​​many exhibit limited design adaptations​​ for extreme tactical environments, including ​​shock resistance, electromagnetic interference mitigation​​, and secure communication, in aspects such as design objectives, certification standards, or product specifications. The main limitations are as follows:

1. Predominance of “Military Adaptation” Over “Military-Specific Development”

Many battlefield ultrasound devices ​​primarily utilize a “civilian-to-military” adaptation approach​​, with ​​relatively few designed​​ through dedicated military-specific processes. Consequently, many systems ​​may exhibit limited incorporation​​ of systematic mechanisms tailored to tactical requirements from the initial development stage. Challenges remain in key domains, including operational interface design, structural robustness, energy supply, and secure communication.

2. Constraints in AI application scope

Current AI applications are ​​mostly focused​​ on image optimization and annotation, while advancements in autonomous diagnostic and task-execution functionalities ​​remain limited​​. As a result, existing systems may have ​​limited capability for​​ independent deployment in remote diagnostic scenarios or seamless integration with unmanned combat units [94].

3. Variability in deployment system standardization

Military equipment models across different countries ​​vary significantly​​, and deployment standards at the unit level are ​​still under development​​. This situation may ​​impede​​ device interoperability and affect the efficiency of cross-domain collaboration [95].

4. Gaps in training mechanisms and data resources

Frontline operators ​​often receive insufficient system training​​, compounded by ​​notable differences among devices​​. Few ‘train-as-you-use’ mechanisms ​​are in place​​. In addition, the limited availability of battlefield imaging datasets may constrain AI model training and limit their generalization capability [81].

5. Insufficient system feedback and evaluation loops

Alwagdani et al. [5] reviewed 35 POCUS studies and reported that, although these devices showed improvements in the efficiency of initial diagnosis in acute and critical care, their adoption has been limited by inconsistent operator proficiency, fluctuations in image quality, and insufficient training mechanisms. These findings ​​indicate gaps​​ in systematic feedback and evaluation loops, which ​​may pose challenges for​​ battlefield deployment.

### Development directions for tactical medical systems

In response to the demands of future multi-domain, high-intensity, and fast-paced combat, battlefield medical systems ​​are expected to evolve​​ from an “independent unit” paradigm toward a “collaborative node” paradigm, ​​through improved integration of​​ diagnosis, decision-making, command, and evacuation. A RAND report ​​outlined that​​ the U.S. future combat casualty care system ​​is shifting toward​​ more intelligent and networked solutions, ​​with an emphasis on​​ AI-assisted decision-making, unmanned supply platforms, modular medical units, and interconnected data networks, which ​​are designed to enhance​​ emergency capabilities in high-threat environments [3]. The “Platinum 10 Minutes” concept ​​further emphasizes the critical importance of​​ rapid diagnosis and resource allocation during the initial treatment window ​​for improving​​ survival outcomes.

Based on this anticipated evolution and operational requirements, the following five development directions are proposed:

1. Strengthen Military-Oriented Ultrasound Design.

A “Battlefield Medical Imaging Special Project” [96] ​​has been proposed​​ under military leadership ​​to advance development and enhance​​ strategic adaptability, ​​with an emphasis on​​ structural design, power supply, image processing, and tactical interaction.

2. Promote Development of a modular medical node system

Development efforts could focus on​​ a unified interface and flexible modular equipment architecture for individual soldiers, squads, vehicle-mounted platforms, and airdropped units [97], ​​to support​​ an expanded role for ultrasound systems in tactical intelligence and medical collaboration.

3. Explore an “Image–AI–Command” closed-loop architecture

Research could investigate​​ a closed-loop process encompassing image acquisition, intelligent prediction, remote consultation, and command coordination through terminals, cloud platforms, and AI-driven central control [98], which ​​would facilitate​​ a transition toward more intelligent, collaborative diagnosis and treatment.

4. Explore a battlefield AI database and intelligent training framework.

A standardized platform for image collection and annotation ​​is worth developing​​, ​​integrating​​ simulation-based training with real-world feedback mechanisms ​​to improve​​ the depth of AI training and ​​enhance​​ frontline operators’ capacity for rapid response in dynamic battlefield environments [99].

5. Encourage International Collaboration and Military–Civil Integration

Active participation in multilateral cooperation platforms ​​is regarded as crucial for​​ advancing standard harmonization and joint exercises [100]. In parallel, joint efforts among universities, defense industries, and AI enterprises ​​are needed to support​​ technology implementation and translational innovation.

## Conclusion

With the increasing prevalence of asymmetric warfare, multi-domain operations, and peacekeeping missions, battlefield medical support systems ​​are expected to evolve toward​​ higher levels of intelligence, modular design, and forward deployment. Ultrasound devices—characterized by radiation-free imaging, portability, and real-time diagnostic capability—are ​​playing an increasingly important role​​ in tactical medicine. This review ​​has evaluated​​ representative battlefield-adapted systems and ​​has identified​​ ongoing challenges related to image quality, environmental adaptability, communication, and operational usability. Current devices continue to face constraints in terms of intelligence, system integration, and standardized deployment, which ​​could constrain​​ their effectiveness in complex operational environments.

Several limitations of this review should be acknowledged. Some devices discussed, such as the Sonosite M-TURBO, ​​represent​​ earlier-generation systems, while others, such as the Butterfly iQ+, ​​exemplify​​ more recent developments ​​with potential applicability​​. Disclosure of equipment deployed in active military contexts is restricted by confidentiality regulations, which ​​constrains​​ the comprehensiveness of this assessment. Despite advances in portability, imaging performance, and AI-assisted functions, trade-offs remain: ruggedized models ​​enhance​​ durability but often ​​at the expense of​​ weight and endurance; ultra-portable devices improve mobility but may compromise imaging quality and battery life; and systems dependent on civilian platforms offer strong telemedicine capabilities but may lack robustness in battlefield conditions. Communication technologies also face ​​constraints​​: conventional wireless protocols ​​are vulnerable to​​ interception and interference; 5G networks ​​depend on​​ infrastructure availability and ​​face​​ cybersecurity risks; Wi-Fi ​​is limited by​​ short range and susceptibility to interference; and satellite-based solutions, while globally accessible, ​​are constrained by​​ power demands, cost, and encryption requirements. In addition, large-scale field studies on ultrasound in military medicine remain ​​scarce​​, ​​highlighting the need for​​ broader empirical validation.

Beyond device-level challenges, several systemic research gaps ​​require attention​​. Evidence heterogeneity and quality ​​pose challenges​​, as available studies ​​vary substantially​​ in design, sample size, endpoints, and operator expertise, with many relying on observational reports that ​​are potentially subject to​​ selection and publication bias. The generalizability of findings ​​is limited​​, since much current evidence is derived from humanitarian or rear-area platforms, whereas data from high-threat forward environments—particularly under hostile electromagnetic conditions or during communication disruptions—remain ​​scarce​​. Differences among devices further complicate evaluation, as some reported performance metrics are based on manufacturer specifications or unblinded assessments, and rigorous head-to-head trials across devices and missions ​​are lacking​​. AI functionalities, although promising, have ​​primarily been evaluated using​​ single-center or offline datasets, with limited prospective validation under frontline conditions such as low bandwidth and unstable power supply. Security and compliance also ​​require further development​​: encryption protocols, zero-trust or role-based access control, and DICOMweb/FHIR interoperability ​​have not been systematically implemented​​ in tactical networks. Finally, confidentiality restrictions on deployed equipment ​​may limit​​ sample representativeness and the transparency of device coverage in the published literature.

Future development ​​should focus on advancing​​ AI-assisted diagnostics, integration of physiological monitoring and communication modules, improved adaptability to extreme environments, enhanced remote collaboration, and ​​deployment​​ on unmanned platforms. At the system level, ​​key priorities could include​​ dedicated research and development, modular deployment, closed-loop architecture design, systematic data resource accumulation, and strengthened international collaboration. Collectively, these measures ​​could represent strategic pathways​​ for advancing the next generation of tactical medical systems. Ultimately, battlefield ultrasound is ​​expected to evolve​​ from a stand-alone imaging device into ​​a key node​​ integrating intelligent perception, diagnostic support, and collaborative command, thereby ​​facilitating the transition​​ of military healthcare from tool-based practice to system-oriented development. The advancement of dedicated military ultrasound systems and their systematic deployment ​​could represent a critical strategic direction​​ for enhancing future combat medical readiness.

## Data Availability

All datasets generated or analyzed in this study are included in the manuscript.

## Abbreviations

AI: Artificial Intelligence
AR: Augmented reality
AUC: Area under the receiver operating characteristic curve
CDT: Continuous data transmission
CDTs: Combining continuous data transmissions
CT: Computed tomography
DICOM: Digital Imaging and Communications in Medicine
ECG: Electrocardiogram
E-FAST: Extended Focused Assessment with Sonography for Trauma
EMC: Electromagnetic compatibility
FAST: Focused Assessment with Sonography for Trauma
FST: Forward surgical teams
IEC: International Electrotechnical Commission
IP: Ingress protection
IVC: Inferior vena cava
MIL-STD-810G: Military Standard 810G
MOOTW: Military operations other than war
MPEG-4: Moving Picture Experts Group Layer 4
POCUS: Point-of-care ultrasound
SpO₂: Oxygen saturation
UGVs: Unmanned ground vehicles
VAS: Visual Analog Scale
WHDs: Wearable health devices
